# Interim Estimates of COVID-19 Vaccine Effectiveness Against COVID-19–Associated Emergency Department or Urgent Care Clinic Encounters and Hospitalizations Among Adults During SARS-CoV-2 B.1.617.2 (Delta) Variant Predominance — Nine States, June–August 2021

**DOI:** 10.15585/mmwr.mm7037e2

**Published:** 2021-09-17

**Authors:** Shaun J. Grannis, Elizabeth A. Rowley, Toan C. Ong, Edward Stenehjem, Nicola P. Klein, Malini B. DeSilva, Allison L. Naleway, Karthik Natarajan, Mark G. Thompson, VISION Network

**Affiliations:** ^1^Center for Biomedical Informatics, Regenstrief Institute, Indianapolis, Indiana; ^2^Westat, Rockville, Maryland; ^3^Department of Medicine, University of Colorado, Anschutz Medical Campus, Aurora, Colorado; ^4^Division of Infectious Diseases and Clinical Epidemiology, Intermountain Healthcare, Salt Lake City, Utah; ^5^Kaiser Permanente Vaccine Study Center, Kaiser Permanente Northern California, Oakland, California; ^6^HealthPartners Institute, Minneapolis, Minnesota; ^7^Center for Health Research, Kaiser Permanente Northwest, Portland, Oregon; ^8^Department of Biomedical Informatics, Columbia University, New York, New York; ^9^CDC COVID-19 Response Team.

Data on COVID-19 vaccine effectiveness (VE) since the B.1.617.2 (Delta) variant of SARS-CoV-2, the virus that causes COVID-19, became the predominant circulating strain in the United States are limited (*1*–*3*). CDC used the VISION Network* to examine medical encounters (32,867) from 187 hospitals and 221 emergency departments (EDs) and urgent care (UC) clinics across nine states during June–August 2021, beginning on the date the Delta variant accounted for >50% of sequenced isolates in each medical facility’s state. VISION Network methods have been published (*4*).

Eligible medical encounters were defined as those among adults aged ≥18 years who had received SARS-CoV-2 molecular testing (primarily reverse transcription–polymerase chain reaction assay within 14 days before or 72 hours after the admission or encounter) and a COVID-19–like illness discharge diagnosis. Vaccination status was documented in electronic health records and immunization registries. Full vaccination was defined as receipt of the second dose of BNT162b2 (Pfizer-BioNTech) or mRNA-1273 (Moderna) mRNA vaccines, or a single dose of Ad26.COV2 (Janssen [Johnson & Johnson]) vaccine ≥14-days before the testing or encounter date. Patients who had received no COVID-19 vaccine doses were considered unvaccinated. Patients who had received 1 mRNA dose only or had received the second dose <14 days before testing or encounter date were excluded. VE was estimated using a test-negative design, calculating the odds of receiving a positive SARS-CoV-2 test result comparing fully vaccinated and unvaccinated patients (referent group). VE was adjusted for age, geographic region, calendar time (days from January 1 to medical event), and virus circulation, and weighted for inverse propensity to be vaccinated or unvaccinated (calculated separately for each VE model). VE estimates with 95% confidence intervals (CIs) that did not overlap were considered statistically different. This activity was reviewed by CDC and was conducted consistent with applicable federal law and CDC policy.^†^

Among fully vaccinated patients, the proportion who had received each vaccine product among hospitalizations and ED/UC encounters, respectively, were Pfizer-BioNTech, 55.3% and 53.6%; Moderna, 38.8% and 36.1%; and Janssen, 6.0% and 10.3%. The median interval from becoming fully vaccinated to the hospital admission or ED/UC encounter, respectively, were 110 and 93 days (Pfizer-BioNTech), 106 and 96 days (Moderna), and 94 and 94 days (Janssen).

Among adults hospitalized with COVID-19–like illness (14,636; median patient age = 65 years, interquartile range [IQR] = 48–77 years), laboratory-confirmed SARS-CoV-2 infections were identified among 18.9% (1,316 of 6,960) of unvaccinated and 3.1% (235 of 7,676) of fully vaccinated patients. Overall, VE against COVID-19 hospitalization was 86% (95% CI = 82%–89%). VE was significantly lower among adults aged ≥75 years (76%) than among those aged 18–74 years (89%) (Table). The difference in VE point estimates between age groups was similar for Pfizer-BioNTech and Moderna vaccines. Across all ages, VE was significantly higher among Moderna vaccine recipients (95%) than among Pfizer-BioNTech (80%) or Janssen (60%) vaccine recipients.

**TABLE:** COVID-19 vaccine effectiveness* against laboratory-confirmed COVID-19–associated emergency department and urgent care clinic encounters and hospitalizations^†^ among adults during SARS-CoV-2 B.1.617.2 (Delta) variant predominance,^§^ by outcome, age group, and vaccine — nine states,^¶^ June–August 2021

Outcome	Total	No. of SARS-CoV-2–positive tests (row %)	VE, % (95% CI)
**All adults (aged ≥18 yrs), any COVID-19 vaccine**			
**COVID-19 hospitalizations**			
Unvaccinated (ref)	6,960	**1,316 (18.9)**	—
Fully vaccinated**	7,676	**235 (3.1)**	86 (82–89)
**COVID-19 ED/UC encounters**			
Unvaccinated (ref)	10,872	**3,145 (28.9)**	—
Fully vaccinated**	7,359	**512 (7.0)**	82 (81–84)
**COVID-19 hospitalizations, any COVID-19 vaccine, by age**			
Age group = 18–74 yrs			
Unvaccinated (ref)	5,708	**1,185 (20.8)**	—
Fully vaccinated**	4,551	**134 (2.9)**	89 (85–92)
Age group = ≥75 yrs			
Unvaccinated (ref)	1,252	**131 (10.5)**	—
Fully vaccinated**	3,125	**101 (3.2)**	76 (64–84)
**COVID-19 hospitalizations by COVID-19 vaccine**			
**BNT162b2 (Pfizer-BioNTech)**			
Unvaccinated (ref)	6,960	**1,316 (18.9)**	—
Fully vaccinated**	4,243	**135 (3.2)**	80 (73–85)
**mRNA-1273 (Moderna)**			
Unvaccinated (ref)	6,960	**1,316 (18.9)**	—
Fully vaccinated**	2,975	**70 (2.4)**	95 (92–97)
**Ad26.COV2.S (Janssen)**			
Unvaccinated (ref)	6,960	**1,316 (18.9)**	—
Fully vaccinated**	458	**30 (6.5)**	60 (31–77)
**COVID-19 ED/UC encounters by COVID-19 vaccine**			
**BNT162b2 (Pfizer-BioNTech)**			
Unvaccinated (ref)	10,872	**3,145 (28.9)**	—
Fully vaccinated**	3,946	**314 (8.0)**	77 (74–80)
**mRNA-1273 (Moderna)**			
Unvaccinated (ref)	10,872	**3,145 (28.9)**	—
Fully vaccinated**	2,656	**98 (3.7)**	92 (89–93)
**Ad26.COV2.S (Janssen)**			
Unvaccinated (ref)	10,872	**3,145 (28.9)**	—
Fully vaccinated**	757	**100 (13.2)**	65 (56–72)

**Abbreviations:** CI = confidence interval; ED = emergency department; HHS = U.S. Department of Health and Human Services; Janssen = Johnson & Johnson vaccine; Ref = referent group; UC = urgent care; VE = vaccine effectiveness.

* VE was estimated using a test-negative design, adjusted for age, geographic region, calendar time (cubic spline with quartile knots), and virus circulation (percentage of SARS-CoV-2–positive results from testing within the counties surrounding the facility on the date of the event) and weighted for inverse propensity to be vaccinated or unvaccinated (calculated separately for each of the 10 VE models) using facility characteristics, sociodemographics, and underlying medical conditions.

^†^ Medical events with a discharge code consistent with COVID-19-like illness were included, such as acute respiratory illness (e.g., COVID-19, respiratory failure, or pneumonia) or related signs or symptoms (cough, fever, dyspnea, vomiting, or diarrhea) using diagnosis codes from the ninth and tenth revisions of the *International Classification of Diseases*. Clinician-ordered molecular assays (e.g., real-time reverse transcription–polymerase chain reaction) for SARS-CoV-2 occurring ≤14 days before to <72 hours after hospital admission or ED/UC encounter were included.

**^§^** Medical events occurred when Delta variant was predominant (>50%) in each state, according to data from network partners, CDC COVID data tracker (https://covid.cdc.gov), and HHS Protects Public Data Hub (https://protect-public.hhs.gov).

^¶^ Partners contributing both ED/UC encounters and hospitalizations in 2021 were in Indiana (range of earliest to latest medical event: July 3–26), Oregon and Washington (June 30–August 4), and Utah (June 1–July 24). Partners contributing hospitalizations only were in California (June 23–August 4), Colorado (June 3–July 20), Minnesota and Wisconsin (July 1–August 2), and New York (June 30–July 25).

** Full vaccination was defined as index testing or admission date ≥14 days after second dose of BNT162b2 (Pfizer-BioNTech) or mRNA-1273 (Moderna) or after a single dose of Ad26.COV2 (Janssen) vaccines.

Among adults with ED/UC encounters for COVID-19–like illness (18,231; median patient age = 43 years, IQR = 29–62 years), laboratory-confirmed SARS-CoV-2 infections were identified among 28.9% (3,145 of 10,872) of unvaccinated and 7.0% (512 of 7,359) of fully vaccinated patients. VE against COVID-19 ED/UC encounters was 82% (95% CI = 81%–84%). VE was highest among Moderna vaccine recipients (92%), followed by Pfizer-BioNTech vaccine recipients (77%), and was lowest (65%) for Janssen vaccine recipients (Table).

In this multistate interim analysis of 32,867 medical encounters among adults of all ages during June–August 2021, when the Delta variant was predominant in the United States, VE of all three authorized COVID-19 vaccines combined remained high against hospitalization (86%) and ED/UC encounters (82%). These overall VE estimates were similar to those during the months before Delta became predominant (*2*,*4*). However, VE against COVID-19 hospitalization among adults aged ≥75 years was significantly lower than that among adults aged <75 years, which had not been observed previously from this data source (*4*). This moderate decline should be interpreted with caution and might be related to changes in SARS-CoV-2, waning of vaccine-induced immunity with increased time since vaccination, or a combination of factors. Differences in VE between the two mRNA vaccines, which had not been observed previously in the VISION Network (*4*), are consistent with another recent finding.^§^ Further examination of the magnitude and sources of product-specific VE differences are also warranted.

The findings in this report are subject to at least three limitations. First, VE by time since vaccination was not examined; further evaluation of possible waning of vaccine protection is currently underway. Second, VE for partial vaccination was not assessed. Finally, although the facilities in this study serve heterogenous populations in nine states, the findings might not be generalizable to the U.S. population.

These findings reaffirm the high protection of COVID-19 vaccines against moderate and severe COVID-19 resulting in ED, UC, and hospital visits and underscore the importance of full COVID-19 vaccination and continued benefits of COVID-19 vaccination during Delta variant predominance. 

## Footnotes

* Funded by CDC, the VISION network includes Columbia University Irving Medical Center (New York), HealthPartners (Minnesota and Wisconsin), Intermountain Healthcare (Utah), Kaiser Permanente Northern California (California), Kaiser Permanente Northwest (Oregon and Washington), Regenstrief Institute (Indiana), and University of Colorado (Colorado).

† 45 C.F.R. part 46; 21 C.F.R. part 56.

§
https://www.medrxiv.org/content/10.1101/2021.08.06.21261707v2
