# *Erratum:* Vol. 70, No. 37

**DOI:** 10.15585/mmwr.mm7049a6

**Published:** 2021-12-10

In the report “Interim Estimates of COVID-19 Vaccine Effectiveness Against COVID-19–Associated Emergency Department or Urgent Care Clinic Encounters and Hospitalizations Among Adults During SARS-CoV-2 B.1.617.2 (Delta) Variant Predominance — Nine States, June–August 2021,” on page 1293, the following statements should have appeared after the author affiliations: “**All authors have completed and submitted the International Committee of Medical Journal Editors form for disclosure of potential conflicts of interest. Shaun J. Grannis reports grants from the Patient-Centered Outcomes Research Institute, Agency for Healthcare Research and Quality, National Institute of Mental Health, National Center for Advancing Translational Sciences, and California Healthcare Foundation; consulting fees from RTI International and Indiana Health Information Exchange; and two U.S. patent applications unrelated to this publication: “Method and system for creating synthetic unstructured free tax medical data for training machine learning models” (#20200035360) and “Predictive Modeling For Health Services” (#20200312457). Nicola P. Klein reports research support from Pfizer for COVID-19 vaccine clinical trials and research support from Pfizer, Merck, GlaxoSmithKline, Sanofi Pasteur, and Protein Sciences (now Sanofi Pasteur) for unrelated studies. Allison L. Naleway reports funding from Vir Biotechnology for research unrelated to this study and Pfizer research funding to Kaiser Permanente Northwest for unrelated study of meningococcal B vaccine safety during pregnancy. No other potential conflicts of interest were disclosed.**”

